# Hyperthermic intraperitoneal chemotherapy for patients with gastric cancer based on laboratory tests is safe: a single Chinese center analysis

**DOI:** 10.1186/s12893-022-01795-6

**Published:** 2022-09-18

**Authors:** Yunzi Wu, Xiaohao Zheng, Chunyang Sun, Shenghui Wang, Shikang Ding, Ming Wu, Jing Zhang, Bingzhi Wang, Liyan Xue, Lin Yang, Yantao Tian, Yibin Xie

**Affiliations:** 1 Department of Pancreatic and Gastric Surgery, National Cancer Center/National Clinical Research Center for Cancer/Cancer Hospital, Chinese Academy of Medical Sciences and Peking Union Medical College, Beijing, China; 2 Department of General Surgery, The Central Hospital of Jia Mu Si City, Jia Mu Si, Hei Long Jiang China; 3 Department of General Surgery, Civil Aviation General Hospital, Beijing, China; 4 Department of Gastrointestinal Surgery, Yun Cheng Center Hospital, Yuncheng, Shanxi China; 5 Department of Abdominal Surgery, Huanxing Cancer Hospital, Beijing, China; 6 Department of Pathology, National Cancer Center/National Clinical Research Center/Cancer Hospital, Chinese Academy of Medical Sciences and Peking Union Medical College, Beijing, China; 7 Department of Medical Oncology, National Cancer Center/National Clinical Research Center/Cancer Hospital, Chinese Academy of Medical Sciences and Peking Union Medical College, Beijing, 100021 China; 8 Department of Pancreatic and Gastric Surgery, National Cancer Center/National Clinical Research Center for Cancer/Hebei Cancer Hospital, Chinese Academy of Medical Sciences, Langfang, 065001 China

**Keywords:** HIPEC, Gastric cancer, Treatment, Surgery, Safety

## Abstract

**Purpose:**

About 15%—40% of gastric cancer patients have peritoneal metastasis, which leads to poor prognosis. Hyperthermic intraperitoneal chemotherapy (HIPEC) is considered to be an effective treatment for these patients. This study evaluated the efficacy and safety of HIPEC in patients with gastric cancer diagnosed from laboratory tests.

**Methods:**

The clinical and pathological data of 63 patients with gastric cancer who underwent HIPEC in 2017–2021 were prospectively recorded. Fifty-five patients underwent cytoreductive surgery + HIPEC, and eight patients received HIPEC alone. The factors associated with HIPEC safety and efficacy were analyzed. The primary endpoint was overall survival.

**Results:**

The average patient age was 54.84 years and 68.3% of patients were male. Moreover, 79.4% of patients had a peritoneal carcinoma index (PCI) score of ≤ 7 and 61.9% had a completeness of cytoreduction score of 0. Because of peritoneal metastasis, 29 patients (46.03%) were classified as stage IV. Laboratory tests showed no differences in pre-HIPEC blood test results compared to post-HIPEC results after removing the effects of surgery. HIPEC treatment did not cause obvious liver or kidney damage. Serum calcium levels decreased significantly after HIPEC (*P* = 0.0018). The Karnofsky performance status (KPS) score correlated with the patient’s physical function and improved after HIPEC (*P* = 0.0045). In coagulation tests, FDP (*P* < 0.0001) and D-dimer (*P* < 0.0001) levels increased significantly and CA242 (*P* = 0.0159), CA724 (*P* < 0.0001), and CEA (*P* < 0.0014) levels decreased significantly after HIPEC. Completeness of cytoreduction score was an independent prognostic factor. HIPEC did not show a survival benefit in patients with gastric cancer (*P* = 0.5505).

**Conclusion:**

HIPEC is a safe treatment for patients with gastric cancer with peritoneal metastasis based on the laboratory tests. However, the efficacy of this treatment on gastric-derived peritoneal metastases requires further confirmation.

**Supplementary Information:**

The online version contains supplementary material available at 10.1186/s12893-022-01795-6.

## Introduction

According to GLOBOCAN newly released data, there were 1,089,103 new patients with gastric cancer and 768,793 deaths in 2020 [1]. In China, there are 403,000 new cases of gastric cancer annually. Gastric cancer is the second most common cancer and the third most common cause of cancer-related death [2]. Peritoneal metastasis from gastric cancer is considered incurable and has a poor prognosis. Although data in Asian populations are lacking, according to the results of studies worldwide, the incidence of peritoneal metastasis in patients with gastric cancer is 15–43% [3]. This indicated that there are at least 60,000 patients with gastric cancer with peritoneal metastasis in China.

Hyperthermic intraperitoneal chemotherapy (HIPEC) is a therapeutic technique that aims to prevent and treat peritoneal cancer (PC) and malignant ascites by heating the perfusate containing chemotherapeutic drugs to the therapeutic temperature and instilling it into the abdominal cavity of patients with tumor for a certain period. Although HIPEC is frequently used in the treatment of peritoneal metastases from abdominal malignancies, such as gastric cancer [4], HIPEC has an irreplaceable role in patients with malignant ascites. A study in China showed that HIPEC significantly improved the overall survival (OS) and treatment of malignant ascites in patients with gastric cancer with initially high peritoneal carcinoma index (PCI) scores and positive peritoneal lavage fluid with peritoneal metastasis (*P* < 0.001) [5]. This result was confirmed by multicenter studies in Spain [6, 7].

The effect of HIPEC on the OS of patients with gastric cancer remains unknown. Some studies have shown that HIPEC cannot prolong the OS of patients with gastric cancer [8]. Diniz et al. performed postoperative HIPEC in patients with gastric cancer after neoadjuvant treatment. The results showed that increasing HIPEC treatment did not improve the OS (*P* = 0.517) or recurrence-free survival (*P* = 0.993), while other studies showed that HIPEC had an advantage in terms of disease-free survival [9]. Reutovich performed HIPEC in patients with gastric cancer with serous invasion. There were no significant differences in complications between the two groups (*P* = 0.254). HIPEC effectively reduced the occurrence of metachronous peritoneal metastasis in such patients (12.8% *vs* 27.6%, *P* < 0.001). The 3-year disease-free survival rate increased (47% *vs* 27%, *P* = 0.0024) [10]. Similarly, Zhong et al*.* showed that HIPEC could prolong the disease-free survival of patients with gastric cancer (*P* = 0.031) [11]. Although several studies have shown that HIPEC did not increase short-term complications, the laboratory test results varied dramatically after HIPEC; however, this change was rarely mentioned. The present study aimed to reveal the safety of HIPEC and the factors influencing OS based on the laboratory test results.

## Materials and methods

### Patients and database

We prospectively recorded the clinical data of patients with gastric cancer who underwent HIPEC between 2017 and 2021. A total of 63 patients were treated with HIPEC. Cancer radical resection (CRR) + HIPEC was performed for patients who have risk factors for peritoneal metastasis but without peritoneal metastases. For patients with peritoneal metastases who can be operated, they received cytoreductive surgery (CRS) + HIPEC. For patients with a large amount of ascites or extensive metastases in the abdominal cavity at the first diagnosis, they received conversion therapy + HIPEC. Of these, 55 underwent CRS or CRR, and 55 patients who did not receive HIPEC were matched for analysis in a ratio of 1:1 according to the TNM stage. Only eight of them received conversion therapy + HIPEC. Thirty-three patients (53%) received neoadjuvant chemotherapy with SOX regimen (S-1 plus oxaliplatin). This study was approved by the ethics committee of the National Cancer Center/National Clinical Research Center for Cancer/Cancer Hospital, Chinese Academy of Medical Sciences, and Peking Union Medical College (NCC 14-067-857, June 16, 2014).

### Peritoneal carcinoma index (PCI)

Using the Peritoneal Carcinoma Index (PCI) Quantitative Assessment of PC Involvement [12], Sugarbaker divided the abdomen into 13 areas, combined with the lesion size (LS) in each area to add and score, as much as possible, detect the number of all tumors invading the peritoneum, and evaluate the degree of PC involvement. In addition to the peritoneum, areas 0–8 also include cancer nodules on the corresponding anatomical structures in this area. For LS scoring, the size of the tumor nodules in each area was measured after dissociating all adhesions and fully exposing the visceral and parietal peritoneal surfaces of the abdominal cavity. The LS score ranged from 0 to 3, with the largest nodule diameter visible to the naked eye as a representative scoring object. LS-0 indicates that no peritoneal lesions were found, LS-1 indicates lesions ≤ 0.5 cm in diameter, LS-2 indicates lesions 0.5–5 cm in diameter, and LS-3 indicates lesions > 5 cm in diameter or confluent lesions. Complete resection of the primary tumor or locally recurrent tumors was not required. Tumor nodules fused into sheets or organs were directly classified as LS-3, even if lamellar fusions were present. The cumulative LS score of each district was the PCI score, and the total score ranged from 0 to 39. The PCI score was evaluated before CRS.

### Completeness of cytoreduction score and KPS

Patients should undergo complete CRS before HIPEC to maximize the removal of macroscopic tumor lesions, and HIPEC can better reduce or remove residual small lesions after CRS. Currently, the completeness of cytoreduction (CC) scoring developed by Sugarbaker is used internationally to evaluate residual tumor size during surgery [13]. CC-0 indicates no macroscopic tumor nodules in the entire abdominal and pelvic cavity after CRS; CC-1 indicates postoperative residual tumor diameter < 0.25 cm; CC-2 indicates residual tumor diameter between 0.25 and 2.5 cm; CC-3 indicates residual tumor diameter > 2.5 cm or unresectable lesions in any part of the abdominal and pelvic cavity. Residual tumors < 0.25 cm in diameter (CC-0 and CC-1) were considered satisfactory CRS. KPS can evaluate the clinical treatment effect through the improvement of quality of life and were also performed in this study.

### HIPEC

(1) Closed method: Perfusion therapy is performed after closing the abdominal cavity. (2) Chemotherapy drugs: Chemotherapy drugs are selected according to the commonly used drugs for intravenous chemotherapy of the primary tumor, past sensitive drugs or drug sensitivity test results, or the patient’s past medical history, disease types and drug characteristics, high tumor tissue penetrability, and molecular weight. Large, low peritoneal absorption rate, synergistic effect with thermal effect, less peritoneal irritation, and tumor effectiveness. (3) Dose of chemotherapy drugs: This refers to the system chemotherapy doses. (4) Perfusion temperature: This was 43 ± 0.1 °C. (5) Perfusion time and frequency: The perfusion time was 60–90 min, usually 60 min. When multiple HIPECs were administered, the interval between treatments was 24 h. (6) Perfusate volume: The effective perfusate was generally 4–6 L, based on the principle of filling the abdominal cavity and smooth circulation. (7) Perfusion speed: This was 400–600 mL/min.

### Laboratory test

Laboratory results were collected on the day before surgery and from all post-HIPEC records. White blood cell (WBC), red blood cell (RBC), hemoglobin (HB), and platelet (PLT) counts were recorded during routine blood tests. ALT, AST, DBIL, GGT, TBIL, IBIL, urea, and CRE levels were used to evaluate liver and kidney function. Electrolyte levels and coagulation status were also analyzed to evaluate the safety. The collection time of preoperative index is the day before operation and the time of postoperative index collection is after all treatment and before discharge. The changes of tumor markers before and after HIPEC treatment can reflect the therapeutic effect to a certain extent.

### Follow-up

Patient follow-up surveys were conducted by clinical specialists. All patients were advised to undergo contrast-enhanced thoracic/abdominal/pelvic computed tomography (CT) and blood tests every 3 months for the first 2 years and every 6 months thereafter. If the patients did not return for follow-up examination at the scheduled time, the follow-up team of our hospital contacted them and recorded the reason. The last follow-up date was January 15, 2022. Surviving patients were recorded on the date of their last follow-up.

### Statistical analysis

Continuous variables are presented as central tendency (mean or median) and dispersion (standard deviation [SD] or interquartile range). For group comparisons of numeric variables, the Student’s t-test was used when data were normally distributed and the Mann–Whitney test for variables in which distribution was not normal. When categorical predictors were compared between groups, we used Pearson’s χ^2^ test or Fisher’s exact test. Survival analysis included the Kaplan–Meier product-limit estimator for the median OS. The two survival curves were compared using the log-rank test. The median follow-up was determined using the reverse Kaplan–Meier method. Cox regression analysis was performed to obtain crude and adjusted hazard ratios for both OS and DFS. The significance level for all tests was reached when the two-tailed P-value was < 0.05. Statistical analyses were performed using the IBM SPSS Statistics (version 26.0) and Prism software (version 9).

## Results

### Clinical parameters of patients with gastric cancer who underwent HIPEC

Our HIPEC cohort consisted of 63 patients with an average age of 54.84 years, and 43 were male (68%). During surgery, we observed that < 25% of patients had ascites, with a maximum of 2000 mL ascites. Fifty (80%) patients had a PCI score ≤ 7 and 39 (62%) had a CC score of 0. Eleven patients (18%) received a three-drug combination of intraperitoneal hyperthermic perfusion therapy. The drug dose was related to the patient’s weight and drug tolerance (Table 1).

**Table 1:** Clinical paraments of gastric cancer patients underwent HIPEC

Clinical paraments	
Age, year (mean ± SD)	54.84 ± 12.84
Gender	
Male	43 (68%)
Female	20 (32%)
BMI, kg/m^2^ (mean ± SD)	22.52 ± 3.57
Ascites	
Yes	15 (24%)
No	48 (76%)
Ascites volume/mL	0–2000
Peritoneal lavage fluid	
Positive	11 (17%)
Negative	6 (10%)
NA	46 (73%)
PCI score	
≤ 7	50 (79%)
> 7	13 (21%)
Cytoreductive surgery	
Yes	55 (87%)
No	8 (13%)
Completeness of cytoreduction score	
0	39 (62%)
1	4 (6%)
2	3 (5%)
3	17 (27%)
The Number of HIPEC	
One	6 (10%)
Two	8 (13%)
Three	45 (72%)
Four	2 (3%)
Five	1 (2%)
HIPEC administration	
Oxaliplatin + Raltitrexed + Lobaplatin	11 (18%)
Oxaliplatin + Raltitrexed	1 (2%)
Lobaplatin	50 (80%)
Oxaliplatin Dose/mg	280–385
Raltitrexed Dose/mg	4–5.5
Lobaplatin Dose/mg	60–350
Neoadjuvant chemotherapy	
Yes	33 (53%)
No	29 (47%)
Surgery method	
Proximal gastrectomy	2 (3%)
Total gastrectomy	33 (52%)
Distal gastrectomy	20 (32%)
Without gastrectomy	8 (13%)
Laparoscopic surgery	
Yes	45 (72%)
No	18 (28%)
Intraoperative blood loss/mL	222.5 ± 210.83

### Pathological parameters of patients with gastric cancer who underwent HIPEC

According to the Borrmann classification, 12 patients had type I and type II, and 26 patients had type III and type IV. The World Health Organization pathological type of the patients was mainly adenocarcinoma, and 24 had signet ring cell carcinoma. Fifty-five patients (87%) had poorly differentiated components. In the Lauren classification, 12 (19%) patients were diagnosed with intestinal type, 21 (33%) had diffuse type, and 11 (18%) had mixed type. Forty-six (73%) patients had neurological invasion at the time of pathological diagnosis, and 41(65%) patients had vascular invasion. All patients were immunohistochemically confirmed to not have uncommon pathological type (Table 2).

**Table 2:** Pathological Paraments of Gastric Cancer Patients Underwent HIPEC

Pathological paraments	
Tumor size, cm	6.18 ± 3.08
Borrmann classification	
Type-I	4 (6%)
Type-II	8 (13%)
Type-III	12 (19%)
Type-IV	14 (22%)
Unknown	25 (40%)
WHO classification	
Adenocarcinoma	55 (87%)
Others	7 (11%)
Unknown	1 (2%)
Signet ring cell carcinoma	
Yes	24 (38%)
No	37 (58%)
Unknown	2 (4%)
Differentiation	
Poor	43 (68%)
Poor-medium	12 (19%)
Medium	3 (5%)
Unknown	5 (8%)
Lauren classification	
Intestinal type	12 (19%)
Diffuse type	21 (33%)
Mixed type	11 (18%)
Unknown	19 (30%)
Nerve invasion	
Yes	46 (73%)
No	5 (8%)
Unknown	12 (19%)
Vascular invasion	
Yes	41 (65%)
No	10 (16%)
Unknown	12 (19%)
T Stage	
T3	7 (11%)
T4	29 (47%)
ypT1a	2 (3%)
ypT2	1 (1%)
ypT3	2 (3%)
ypT4	14 (22%)
Unknown	8 (13%)
N Stage	
N0	4 (7%)
N1	1 (1%)
N2	11 (13%)
N3	20 (33%)
ypN0	5 (8%)
ypN1	3 (6%)
ypN2	3 (6%)
ypN3	7 (11%)
Unknown	9 (15%)
M Stage	
0	40 (65%)
1	20 (32%)
Unknown	3 (6%)
TNM Stage	
Stage I	3 (6%)
Stage II	2 (3%)
Stage III	37 (58%)
Stage IV	19 (30%)
Unknown	2 (3%)
Detected lymph nodes number	36 ± 17.66
Positive lymph nodes number	11.89 ± 14.99
AFP	
Positive	0 (0%)
Negative	32 (51%)
Unknown	31 (49%)
GPC3	
Positive	2 (3%)
Negative	28 (45%)
Unknown	33 (52%)
SALL4	
Positive	5 (8%)
Negative	26 (41%)
Unknown	32 (51%)
Her2	
Negative	29 (46%)
+	14 (22%)
2 +	2 (3%)
3 +	1 (2%)
Unknown	17 (27%)

### Laboratory test proves safety of HIPEC in patients with gastric cancer

**Fig. 1:**
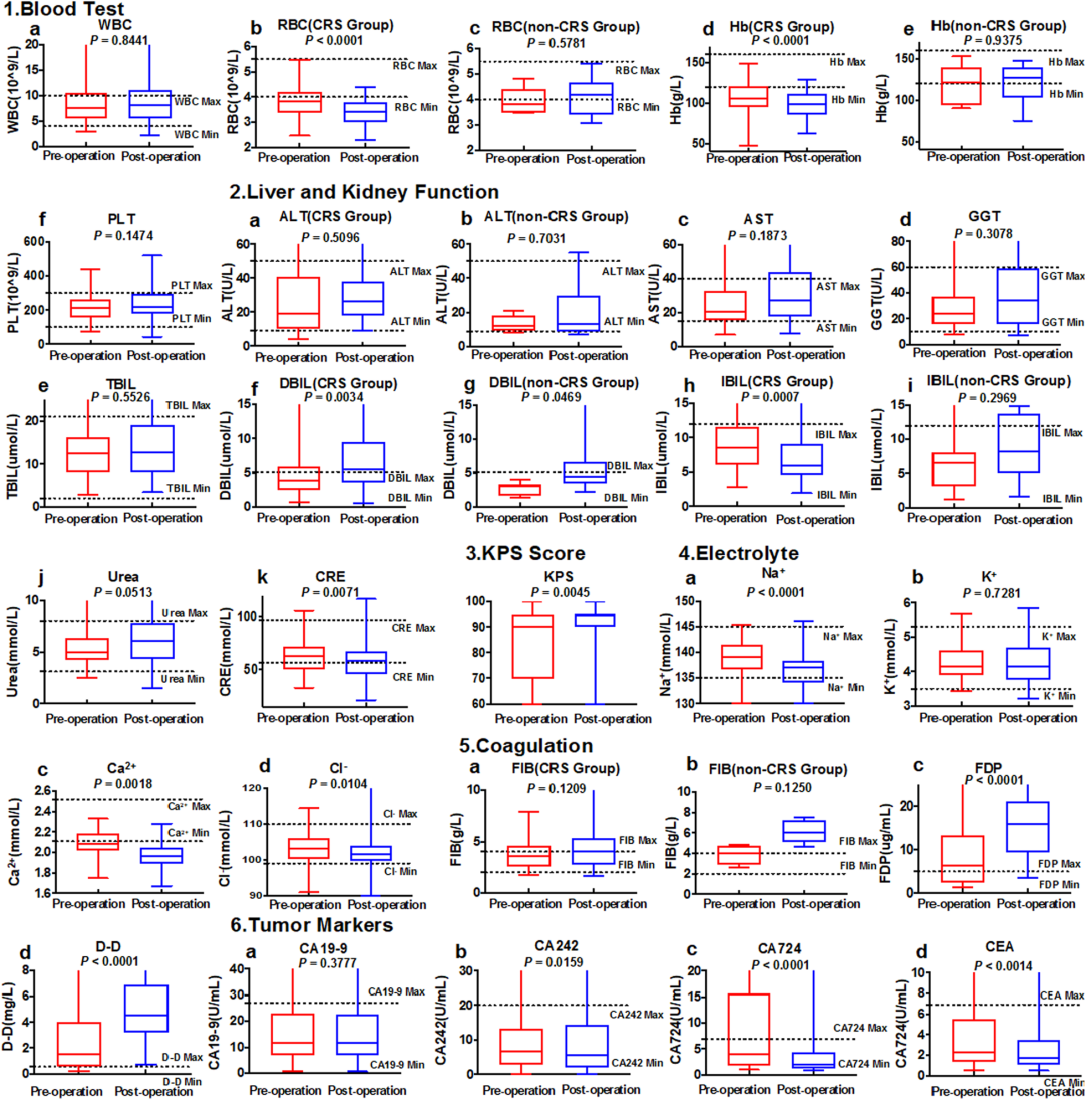
Laboratory tests of patients who underwent HIPEC. 1**a** WBC; 1**b**, **c** RBC; 1**d**, **e**: Hb; 1**f** PLT; **2a**, **b** ALT; **2c** AST; **2d** GGT; **2e** TBIL; **2f**, **g** DBIL; **2 h**, **i** IBIL; **j** Urea; **k** CRE; **3** KPS; **4a** Na^+^; **4b** K^+^; **4c** Ca^2+^; **4d** Cl^−^; **5a**, **b**: FIB; **5c** FDP; **5d** D-Dimer; **6a** CA19-9; **6b** CA242; **6c** CA724; **6d** CEA

Patients were divided into two groups based on whether they underwent CRS. To exclude the effect of surgery on HIPEC, we compared the blood test indices between the two groups, and the results are presented in Additional file 1: Table S1. The blood test results before and after HIPEC are shown in Fig. 1. There were no significant differences in WBC count (*P* = 0.8441) and PLT count (*P* = 0.1474), while, in the CRS group, we observed that RBC (*P* < 0.0001) and HB (*P* < 0.0001) counts decreased after CRS, while, in the non-CRS cohort, these factors did not decrease (Fig. 1a). In liver and kidney function tests, before and after HIPEC, there were no significant differences in ALT (*P* = 0.5096), AST (*P* = 0.1873), GGT (*P* = 0.3078), TBIL (*P* < 0.5526), and urea (*P* = 0.0513), while DBIL (*P* = 0.0034 and *P* = 0.0469), and IBIL significantly increased (*P* = 0.0007). KPS scores improved after HIPEC (*P* = 0.0045) (Fig. 1c). Na^+^ (*P* < 0.0001), Ca^2+^ (*P* = 0.0018), and Cl^−^ (*P* < 0.0104) levels changed after HIPEC, and there was no difference in K^+^ (*P* = 0.7281). Na^+^ and Cl^−^ levels were within the normal range, while Ca^2+^ levels were lower than normal after treatment, which may have caused hypocalcemia (Fig. 1d). In the coagulation test, FDP (*P* < 0.0001) and D-dimer (*P* < 0.0001) levels were significantly increased in the resection group (Fig. 1e). The CA242 (*P* = 0.0159), CA724 (*P* < 0.0001), and CEA (*P* < 0.0014) levels significantly decreased after HIPEC.

### Univariate and multivariate analysis of prognostic factors in patients who underwent HIPEC

**Fig. 2:**
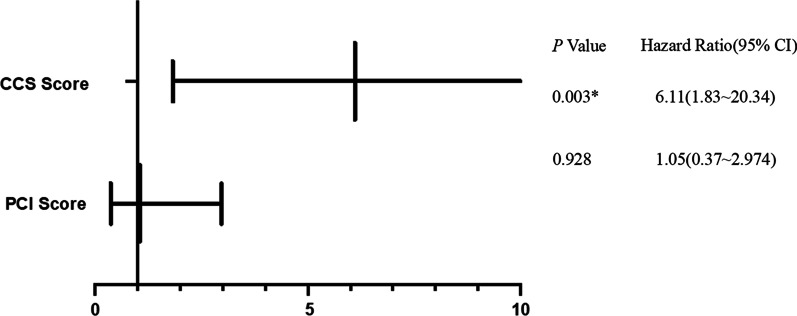
Multivariate analysis of prognostic factors in patients who underwent HIPEC

Clinicopathological factors and laboratory test indices of the patients were included in the prognostic model (Additional file 2: Table S2). Only the PCI (HR = 2.97, 95% CI: 1.21 ~ 7.34) (*P* = 0.018) and CCS scores (HR = 7.13, 95% CI: 2.51 ~ 20.25) (*P* < 0.0001) were prognostic factors in the univariate analysis. In the multivariate analysis, the CCS score (HR = 6.11, 95% CI: 1.83 ~ 20.34) (*P* = 0.003) was an independent prognostic factor (Fig. 2).

### Survival analysis of patients who underwent HIPEC

**Fig. 3:**
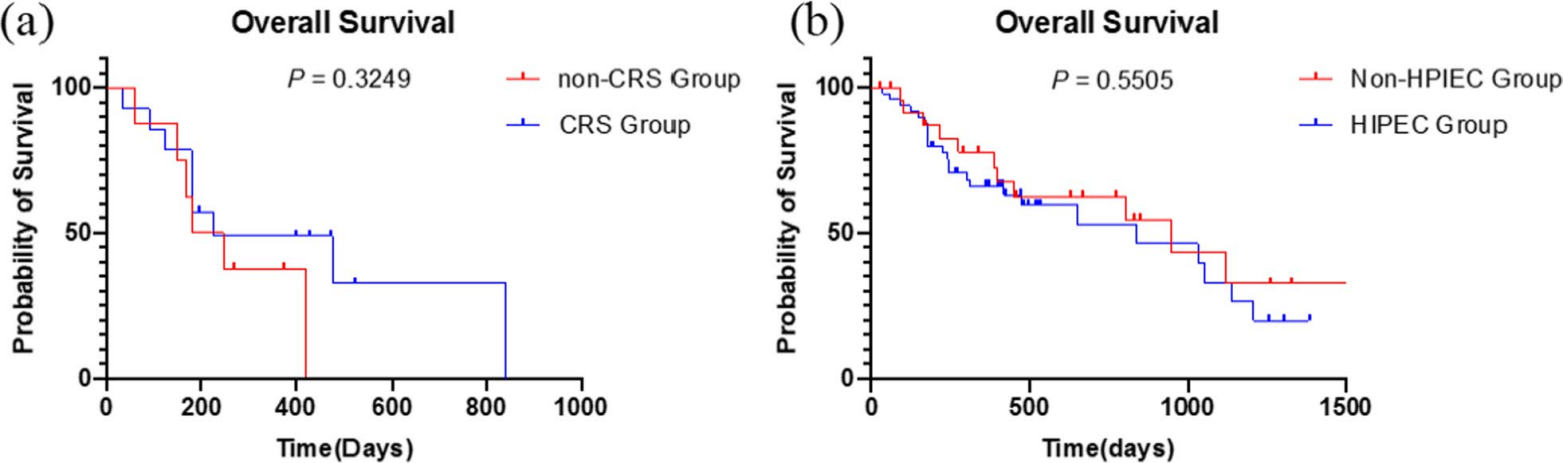
Survival analysis of patients who underwent HIPEC. **a** Difference in survival between patients who underwent and did not underwent surgery. **b** Difference in survival between patients who received and did not receive HIPEC

Tumor resection is important in the treatment of patients. Survival was compared between patients who underwent CRS and those who did not (Fig. 3a). The results showed that the survival of patients who underwent CRS was same as that of patients who did not (*P* = 0.3249). What’s more, to clarify the role of HIPEC in patients, the matched comparison results are presented in Additional file 3: Table S3. The difference in survival between patients who underwent HIPEC and those who did not after resection was not significant (*P* = 0.5505) (Fig. 3b).

### Information on long-term survival of patients with HIPEC

**Fig. 4:**
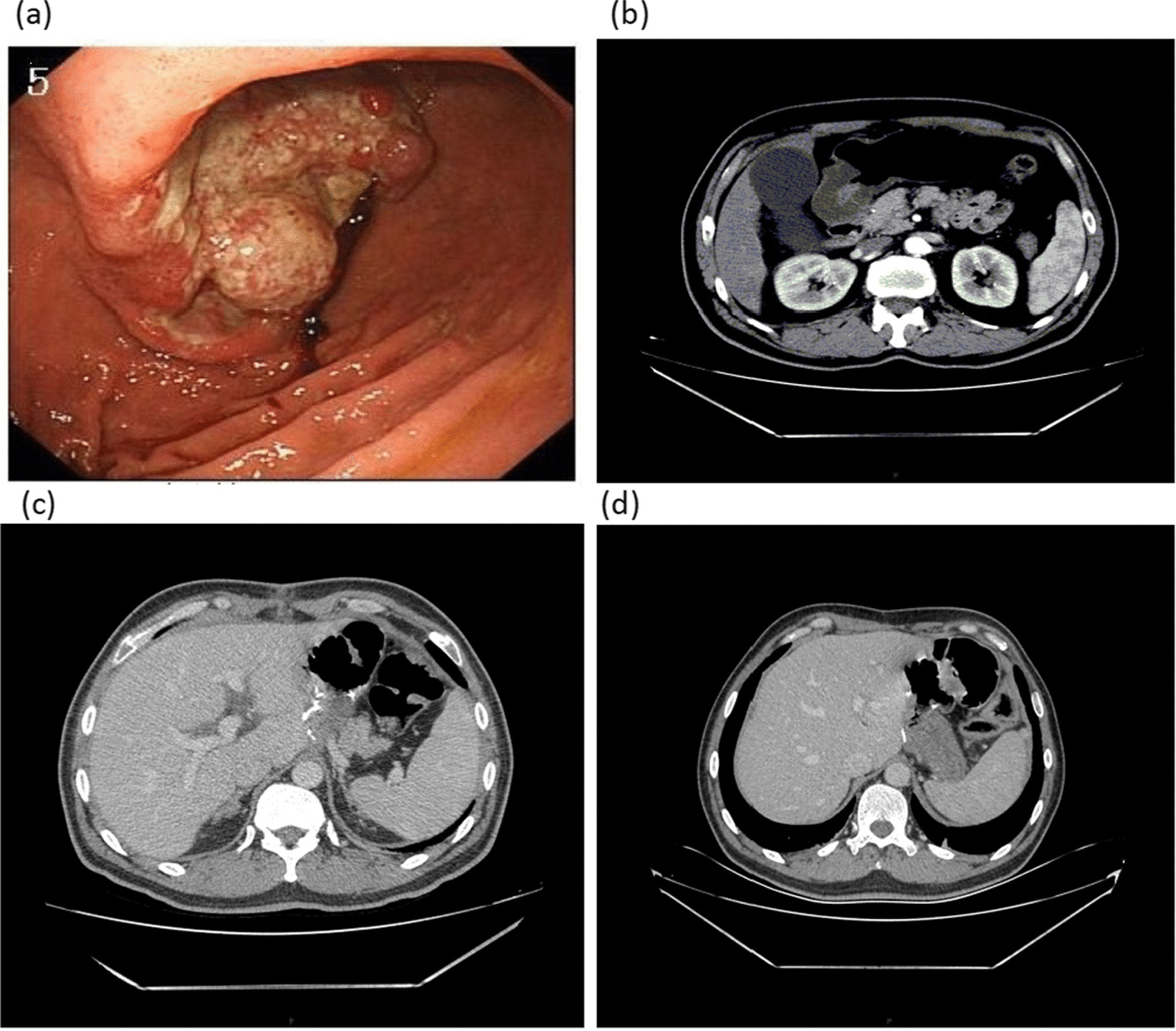
Representative cases of long-term survivors. **a** The patient’s tumor was located in the gastric body before treatment. **b** Patient after neoadjuvant therapy. **c** Postoperative condition of patients. **d** Patient’s recent CT results

Six patients had a survival period of > 1000 days as long-term survivors (Additional file 4: Table S4). Figure 4 shows the data of a long-term survivors with positive peritoneal lavage fluid. Preoperative endoscopy revealed that the tumor was located in the body of the stomach (Fig. 4a). The tumor size decreased in patients who received neoadjuvant therapy (Fig. 4b). The patient’s tumor was completely resected after surgery (Fig. 4c). The patient’s tumor did not recur during recent CT examinations (Fig. 4d).

## Discussion

Peritoneal metastasis is common in patients with gastric cancer. Patients with gastric cancer and peritoneal metastases have poor prognosis. In the multidisciplinary treatment of gastric cancer, postoperative adjuvant chemotherapy and radiotherapy have limited effects on the treatment of peritoneal metastases [14]. Although peritoneal metastasis can be determined by CT and presence of ascites, it is easy to ignore smaller peritoneal metastases. Currently, the only way to determine whether peritoneal metastases have occurred is to perform laparoscopic exploration during surgery [15]. HIPEC is not a routine and easily achievable treatment method because it requires professional equipment and standardized treatment team.

The greatest limitation of our study is the small number of cases. Some of our patients received neoadjuvant therapy, and staging covers stage I to stage IV, which increases the complexity in our patients. Peritoneal lavage cytology is used as a staging factor in patients with gastric cancer. Patients with positive cytology before surgery have poor OS (*P* < 0.0001), and neoadjuvant chemotherapy with positive cytology can turn into negative cytology, which is correlated with OS improvement (*P* < 0.0001) [16]. However, most of our patients were not tested for peritoneal lavage cytology, which is a limitation of our study.

As a developing technology, the safety of HIPEC has been confirmed by many researchers in terms of short-term postoperative complications [8, 17–21]. Overall, HIPEC for gastric cancer did not increase the incidence of complications in patients with gastric cancer. HIPEC is irreplaceable as a special treatment. A more effective evaluation of HIPEC’s safety under the condition of strictly mastering the indications is required to widely increase the use of HIPEC and lay a foundation for clarifying the prognostic correlation of HIPEC as soon as possible. Based on this, our study mainly determined the safety of HIPEC based on the hematological examination status of patients before and after HIPEC.

In our patients, HIPEC may have affected the patients’ electrolyte levels. Our study differs greatly from other studies. They only focused on short-term postoperative complications, such as anastomotic leakage and postoperative bleeding. We analyzed the safety of HIPEC from the perspective of laboratory inspection. The advantage of our study is that we obtained objective indicators of change. WBC levels in our patient before and after HIPEC were within the normal range, indicating that HIPEC did not increase the risk of infection. The decrease in HB and RBC counts in patients is mainly due to blood loss during surgical resection. The patient’s liver and kidney functions were almost normal after HIPEC. Even if IBIL, DBIL, and CRE change, their mean values are within the normal range, indicating that HIPEC does not cause liver and kidney injuries. This has also been observed in other studies [22]. Electrolyte abnormalities are not observed only in our patients who received HIPEC [23]. Our study demonstrated the need to monitor hypocalcemia in patients.

Overall, HIPEC remains an extremely safe treatment modality from a laboratory perspective. From the point of view of laboratory examination, after comprehensive treatment of the tumor, the patient’s tumor marker levels decreased. According to the KPS score, our patients recovered within a short period after treatment. Although the impact of HIPEC on prognosis was not observed in our study, HIPEC still plays an irreplaceable role in patients with gastric cancer. A study in China showed that HIPEC significantly improved the OS and treatment of malignant ascites in patients with gastric cancer with initially higher PCI scores and positive peritoneal lavage fluid with peritoneal metastasis (*P* < 0.001) [5]. This was also confirmed by a multicenter study in Spain [6, 7].

Presently, for indicators related to the prognosis of patients with gastric cancer treated with HIPEC, international studies mainly reported that the PCI score and whether to accept CRS are the key factors [24–26]. Ji et al. believe that postoperative CC score is a prognostic factor, and the prognosis of patients with CCS-0 is good [27]. Many studies have shown that the PCI score can be used as an independent prognostic factor, and patients with a score < 7 can improve their prognosis after thermal perfusion [28–30].

Our findings are similar, through Cox regression analysis of multiple clinicopathological factors. In the univariate analysis, PCI score (*P* = 0.018) and CCS score (*P* < 0.0001) scores were prognostic factors. Multivariate Cox regression analysis showed that only the CCS score (*P* = 0.018) was an independent prognostic factor. This indicates that, even if there is a certain peritoneal metastasis in patients with gastric cancer, peritoneal metastasis and primary tumor should be resected as much as possible.

Some patients with gastric cancer with long-term survival were observed in our cohort. Their common feature was low PCI score (< 7); all patients underwent CRS and had CC-0. This indicates that, even if the patient finds that the tumor is already locally advanced, there is still a possibility of recovery after multidisciplinary treatment. It is worth noting that patients require periodic follow-up after treatment. In the dynamic assessment of a medical team, the medical problems that arise are controlled and corrected over time. Under such medical care, the OS of patients is prolonged.

Overall, the use of drugs for the treatment of HIPEC is still based on empirical medication and needs to be evaluated using specific pharmacological methods [4]. It is hoped that large, capable treatment centers can conduct prospective randomized controlled clinical trials of multiple combinations to demonstrate the effectiveness of HIPEC. Neoadjuvant HIPEC and pressurized intraperitoneal aerosol chemotherapy as new treatment methods are worthy of further research [31]. New treatment technologies and methods are widely used, and it is believed that a better judgment of their effects can be obtained through the sorting and analysis of clinical data. In this way, it can benefit patients.

## Conclusion

HIPEC is a safe treatment for patients with gastric cancer with peritoneal metastasis based on the laboratory tests. However, the efficacy of this treatment on gastric-derived peritoneal metastases requires further confirmation.

## Supplementary Information

**Additional file 1: Table S1.** Preoperative and postoperative blood test betweeen CRS/non-CRS Group.

**Additional file 2: Table S2.** Univariate and multivariate analysis of prognostic factors in patients who underwent HIPEC.

**Additional file 3: Table S3.** Baseline Characteristics of HIPEC and gastric cancer patients after 1：1 matched.

**Additional file 4: Table S4.** Clinical characters of long-time survivors.

## Data Availability

The datasets generated during and/or analyzed during the current study are available from the corresponding author on reasonable request.
