# Supplementary material for: Hyperthermic intraperitoneal chemotherapy for patients with gastric cancer based on laboratory tests is safe: a single Chinese center analysis
Source: BMC Surg. 2022 Sep 18;22:342. doi: 10.1186/s12893-022-01795-6 (PMC9482732; doi:10.1186/s12893-022-01795-6)
Supplement: Supplementary file 1 — Additional file 1: Table S1. Preoperative and postoperative blood test betweeen CRS/non-CRS Group. [file 12893_2022_1795_MOESM1_ESM.docx]

| Additional file 1: Table S1 Preoperative and Postoperative Blood Test betweeen CRS/non-CRS Group | | | | | |
| --- | --- | --- | --- | --- | --- |
|  |  | Tumor Biomarkers | CRS Group | Non-CRS Group | *P* Value |
| Tumor Biomarkers | Preoperative | CEA(U/L) | 1.30~6.51 | 1.44~3.08 | 0.7741 |
|  |  | CA199(U/L) | 6.99~21.06 | 6.93~117.00 | 0.3645 |
|  |  | CA242(U/L) | 2.52～12.98 | 3.10～53.10 | 0.5591 |
|  |  | CA724(U/L) | 1.61～14.48 | 2.05～24.60 | 0.311 |
|  | Postoperative | CEA(U/L) | 1.02～3.32 | 1.64 ～26.89 | 0.2136 |
|  |  | CA199(U/L) | 6.45～22.42 | 11.66～100.8 | 0.1629 |
|  |  | CA242(U/L) | 1.63～14.28 | 6.00～44.45 | 0.2208 |
|  |  | CA724(U/L) | 1.17 ～4.37 | 1.66～152.10 | 0.303 |
|  | Change | CEA(U/L) | -0.04～1.96 | -16.25～1.08 | 0.0388* |
|  |  | CA199(U/L) | -2.20～4.68 | -88.66～46.86 | 0.7044 |
|  |  | CA242(U/L) | -0.28～3.17 | -20.83～1.83 | 0.073 |
|  |  | CA724(U/L) | 0.37～11.16 | -22.45～-0.25 | 0.0003* |
| Complete Blood Counts | Preoperative | WBC(10^9/L) | 5.44～10.79 | 4.7～8.94 | 0.6951 |
|  |  | RBC(10^12/L) | 3.37～4.19 | 3.49～4.41 | 0.683 |
|  |  | Hb(g/L) | 95.00～121.00 | 94.00～140.00 | 0.2035 |
|  |  | PLT(10^9/L) | 154.00 ～261.00 | 200.00～283.00 | 0.316 |
|  | Postoperative | WBC(10^9/L) | 5.80～11.19 | 5.08～12.59 | 0.9416 |
|  |  | RBC(10^12/L) | 2.28~4.37 | 3.06~5.4 | 0.0005* |
|  |  | Hb(g/L) | 63~129 | 75~148 | 0.0005* |
|  |  | PLT(10^9/L) | 43～521 | 128～363 | 0.7961 |
|  | Change | WBC(10^9/L) | -3.15～2.59 | -1.64 ～5.89 | 0.2009 |
|  |  | RBC(10^12/L) | -0.67～-0.08 | -0.48 ～0.73 | 0.0301* |
|  |  | Hb(g/L) | -18.00～-2.00 | -15.00 ～19.00 | 0.0775 |
|  |  | PLT(10^9/L) | -32.00～65.00 | -55.00～44.00 | 0.4029 |
| Blood Chemistry | Preoperative | TBIL(μmol/L) | 8.20～16.40 | 4.50～11.30 | 0.0715 |
|  |  | DBIL(μmol/L) | 2.46～5.88 | 1.63～3.31 | 0.036* |
|  |  | IBIL(μmol/L) | 6.12～11.62 | 3.09～13.13 | 0.1373 |
|  |  | ALT(U/L) | 10.00～41.00 | 9.20 ～18.00 | 0.1559 |
|  |  | AST(U/L) | 15.60～36.20 | 15.00～18.50 | 0.0699 |
|  |  | ALP(U/L) | 57.00～80.00 | 66.38～98.03 | 0.1754 |
|  |  | CRE(μmol/L) | 50.00 ～71.00 | 52.00～75.90 | 0.7316 |
|  |  | Urea(mmol/L) | 4.20～6.30 | 4.50 ～6.40 | 0.8575 |
|  | Postoperative | TBIL(μmol/L) | 8.00～17.80 | 7.59～20.48 | 0.8475 |
|  |  | DBIL(μmol/L) | 3.51～ 9.52 | 3.46～6.68 | 0.4584 |
|  |  | IBIL(μmol/L) | 4.49～9.05 | 5.08 ～13.80 | 0.2639 |
|  |  | ALT(U/L) | 18.00～38.00 | 8.75～29.50 | 0.0233* |
|  |  | AST(U/L) | 19.00～45.00 | 13.50～29.50 | 0.053 |
|  |  | ALP(U/L) | 47.00～82.00 | 73.00～90.00 | 0.2767 |
|  |  | CRE(μmol/L) | 46.00～66.00 | 39.00～98.00 | 0.2075 |
|  |  | Urea(mmol/L) | 4.20～7.50 | 5.30～10.80 | 0.1431 |
|  | Change | TBIL(μmol/L) | -4.78～4.00 | -1.04～8.58 | 0.1571 |
|  |  | DBIL(μmol/L) | -1.17～4.60 | 0.19～3.70 | 0.5156 |
|  |  | IBIL(μmol/L) | -4.30～0.67 | -1.48 ～5.60 | 0.0266* |
|  |  | ALT(U/L) | -14.90～14.00 | -0.10～24.80 | 0.7897 |
|  |  | AST(U/L) | -12.30～27.00 | -3.00～12.50 | 0.9522 |
|  |  | ALP(U/L) | -32.0～13.00 | -17.00 ～28.50 | 0.6667 |
|  |  | CRE(μmol/L) | -12.00～3.00 | -7.00～18.83 | 0.0914 |
|  |  | Urea(mmol/L) | -1.30～2.60 | 0.08～3.95 | 0.2648 |
| Electrolytes | Preoperative | Na+(mmol/L) | 136.6～141.4 | 136.0～142.5 | 0.8657 |
|  |  | K+(mmol/L) | 3.84～4.62 | 3.94～4.78 | 0.2286 |
|  |  | Cl-(mmol/L) | 100.30～106.00 | 102.10～104.70 | 0.7806 |
|  |  | Ca2+(mmol/L) | 2.01～2.16 | 2.05～2.25 | 0.319 |
|  | Postoperative | Na+(mmol/L) | 134.0～138.40 | 133.20～138.30 | 0.5731 |
|  |  | K+(mmol/L) | 3.21～5.25 | 3.37～5.85 | 0.2028 |
|  |  | Cl-(mmol/L) | 100.00～104.00 | 99.75～103.20 | 0.5798 |
|  |  | Ca2+(mmol/L) | 1.89～2.04 | 1.87～2.20 | 0.2673 |
|  | Change | Na+(mmol/L) | -5.00～1.10 | -5.40 ～-0.30 | 0.6046 |
|  |  | K+(mmol/L) | -0.43～0.26 | -0.28～0.74 | 0.0837 |
|  |  | Cl-(mmol/L) | -5.00～1.30 | -2.90 ～-0.70 | 0.5743 |
|  |  | Ca2+(mmol/L) | -0.26～0.01 | -0.44～0.14 | 0.5106 |
| Blood Coagulation | Preoperative | D-Dimer(mg/L) | 0.60～4.14 | 0.39～1.72 | 0.1326 |
|  |  | FDP(μg/L) | 2.27～14.04 | 2.22～5.64 | 0.1585 |
|  |  | FIB(g/L) | 2.58～4.56 | 2.90～4.76 | 0.5523 |
|  | Postoperative | D-Dimer(mg/L) | 3.47～7.01 | 1.57～6.29 | 0.2219 |
|  |  | FDP(μg/L) | 10.70～23.91 | 5.09～17.21 | 0.1169 |
|  |  | FIB(g/L) | 2.78～5.34 | 5.15～7.26 | 0.013* |
|  | Change | D-Dimer(mg/L) | 0.85 ～4.74 | 0.20～6.35 | 0.9999 |
|  |  | FDP(μg/L) | 3.81～13.23 | 0.27～15.60 | 0.6916 |
|  |  | FIB(g/L) | -0.65～1.57 | 1.90～3.32 | 0.0145 |
