# Supplementary material for: Hyperthermic intraperitoneal chemotherapy for patients with gastric cancer based on laboratory tests is safe: a single Chinese center analysis
Source: BMC Surg. 2022 Sep 18;22:342. doi: 10.1186/s12893-022-01795-6 (PMC9482732; doi:10.1186/s12893-022-01795-6)
Supplement: Supplementary file 2 — Additional file 2: Table S2. Univariate and multivariate analysis of prognostic factors in patients who underwent HIPEC. [file 12893_2022_1795_MOESM2_ESM.docx]

Additional file 2: Table S2. Univariate and multivariate analysis of prognostic factors in patients who underwent HIPEC

|  | Univariate logistics analysis | | | Multivariate logistics analysis | | |
| --- | --- | --- | --- | --- | --- | --- |
| Characteristics | HR | 95%CI | *P* Value | HR | 95%CI | *P* Value |
| **Gender** |  |  |  |  |  |  |
| Male | Reference |  |  |  |  |  |
| Female | 0.89 | 0.37~2.13 | 0.791 |  |  |  |
| **Age** |  |  |  |  |  |  |
| <60 | Reference |  |  |  |  |  |
| ≥60 | 0.75 | 0.33～1.67 | 0.485 |  |  |  |
| **Neoadjubant Therapy** |  |  |  |  |  |  |
| Yes | Reference |  |  |  |  |  |
| No | 1.217 | 0.54~2.72 | 0.632 |  |  |  |
| **BMI** |  |  |  |  |  |  |
| Normal(18~24) | Reference |  |  |  |  |  |
| Abnormal(<18 OR >24) | 1.286 | 0.58~2.87 | 0.539 |  |  |  |
| **Ascites** |  |  |  |  |  |  |
| Yes | Reference |  |  |  |  |  |
| No | 0.6 | 0.25~1.46 | 0.264 |  |  |  |
| **PCI Score** |  |  |  |  |  |  |
| ≤7 | Reference |  |  | Reference |  |  |
| >7 | 2.97 | 1.21~7.34 | 0.018* | 1.05 | 0.37~2.974 | 0.928 |
| **CCS Score** |  |  |  |  |  |  |
| 0 | Reference |  |  | Reference |  |  |
| >0 | 7.13 | 2.51~20.25 | <0.0001* | 6.11 | 1.83~20.34 | 0.003* |
| **WHO Classification** |  |  |  |  |  |  |
| Adenocarcinoma | Reference |  |  |  |  |  |
| Others | 0.8 | 0.18~3.47 | 0.761 |  |  |  |
| **Borrmann Classification** |  |  |  |  |  |  |
| Type I and II | Reference |  |  |  |  |  |
| Type III and IV | 2.33 | 0.67~8.16 | 0.186 |  |  |  |
| **Lauren Classification** |  |  |  |  |  |  |
| Intestinal Type | Reference |  |  |  |  |  |
| Others | 0.69 | 0.24～2.01 | 0.497 |  |  |  |
| **Diffusion Type** |  |  |  |  |  |  |
| Low | Reference |  |  |  |  |  |
| Others | 0.98 | 0.38～2.52 | 0.967 |  |  |  |
| **signet-ring cell carcinoma** | |  |  |  |  |  |
| Yes | Reference |  |  |  |  |  |
| No | 1.07 | 0.43～2.65 | 0.885 |  |  |  |
| **Nerve Invasion** |  |  |  |  |  |  |
| Yes | Reference |  |  |  |  |  |
| No | 0.62 | 0.14～2.80 | 0.534 |  |  |  |
| **Vascular invasion** |  |  |  |  |  |  |
| Yes | Reference |  |  |  |  |  |
| No | 0.29 | 0.06～1.34 | 0.113 |  |  |  |
| **TNM Stage** |  |  |  |  |  |  |
| Stage I and II | Reference |  |  |  |  |  |
| Stage III and IV | 2.11 | 0.28～15.99 | 0.471 |  |  |  |
