# Supplementary material for: Hyperthermic intraperitoneal chemotherapy for patients with gastric cancer based on laboratory tests is safe: a single Chinese center analysis
Source: BMC Surg. 2022 Sep 18;22:342. doi: 10.1186/s12893-022-01795-6 (PMC9482732; doi:10.1186/s12893-022-01795-6)
Supplement: Supplementary file 3 — Additional file 3: Table S3. Baseline Characteristics of HIPEC and gastric cancer patients after 1：1 matched. [file 12893_2022_1795_MOESM3_ESM.docx]

Additional file 3: Table S3. Baseline Characteristics of HIPEC and gastric cancer patients after 1：1 matched

|  | **HIPEC Group(n=48)** | **non-HIPEC Group(n=48)** | **P Value** |
| --- | --- | --- | --- |
| **Age (year)** | 55.96±13.38 | 57.04±9.737 | 0.6512 |
| **Gender** |  |  | 0.8321 |
| Male | 31(64.6%) | 30(62.5%) |  |
| Female | 17(35.4%) | 18(37,5%) |  |
| **BMI (kg/m2)** | 22.56±3.404 | 23.88±4.200 | 0.0952 |
| **Neoadjuvant therapy** |  |  | >0.9999 |
| Yes | 25(52.1%) | 25(52.1%) |  |
| No | 23(47.9%) | 23(47.9%) |  |
| **T Stage** |  |  | >0.9999 |
| T1 and T2 | 1(0.02%) | 1(0.02%) |  |
| T3 and T4 | 33(69.0%) | 33(69.0%) |  |
| yp T1 and yp T2 | 2(0.04%) | 2(0.04%) |  |
| yp T3 and yp T4 | 12(25.0%) | 12(25.0%) |  |
| **N Stage** |  |  | >0.9999 |
| N0 and N1 | 5(10.5%) | 5(10.5%) |  |
| N2 and N3 | 29(60.3%) | 29(60.3%) |  |
| yp N0 and yp N1 | 7(14.6%) | 7(14.6%) |  |
| yp N2 and yp N3 | 7(14.6%) | 7(14.6%) |  |
| **M Stage** |  |  | >0.9999 |
| M0 | 41(85.4%) | 41(85.4%) |  |
| M1 | 7(14.6%) | 7(14.6%) |  |
| **TNM Stage** |  |  | >0.9999 |
| Stage I and II | 5(10.5%) | 5(10.5%) |  |
| Stage III and IV | 43(89.5%) | 43(89.5%) |  |
