# Supplementary material for: Hyperthermic intraperitoneal chemotherapy for patients with gastric cancer based on laboratory tests is safe: a single Chinese center analysis
Source: BMC Surg. 2022 Sep 18;22:342. doi: 10.1186/s12893-022-01795-6 (PMC9482732; doi:10.1186/s12893-022-01795-6)
Supplement: Supplementary file 4 — Additional file 4: Table S4. Clinical characters of long-time survivors. [file 12893_2022_1795_MOESM4_ESM.docx]

Additional file 4: Table S4. Clinical characters of long-time survivors.

| Long-term survivors (more than 1000 days) | | | | | | | | | | |
| --- | --- | --- | --- | --- | --- | --- | --- | --- | --- | --- |
| ID | age | Gender | PCI Score | CCS | Neoadjuvant Chemotherapy | Neoadjuvant time | Sugery Method | HIPEC Administration | Status | Overall Survival(days） |
| 1 | 64 | Male | 0 | 0 | No | No | total gastrectomy | Oxaliplatin(350mg)+Letitrexed(4mg) | Death | 1207 |
| 2 | 56 | Male | 0 | 0 | Oxaliplatin 150mg+Tegafur 100mg | 4 | Distal gastrectomy | Oxaliplatin(385mg)+Letitrexed(NA)+Lobaplatin(80mg) | Alive | 1384 |
| 3 | 67 | Female | 0 | 0 | No | No | total gastrectomy | Oxaliplatin(350mg)+Letitrexed(5.5mg)+Lobaplatin(60mg) | Alive | 1254 |
| 4 | 42 | Male | 0 | 0 | Paclitaxel Liposome240mg+Oxaliplatin 170mg+Tegafur 60mg | 3 | total gastrectomy | Oxaliplatin(375mg)+Letitrexed(5.5mg)+Lobaplatin(81mg) | Death | 1051 |
| 5 | 67 | Female | 0 | 0 | FOLFOX4 | 4 | total gastrectomy | Oxaliplatin(280mg)+Letitrexed(4.2mg)+Lobaplatin(80mg) | Alive | 1303 |
| 6 | 35 | Female | 0 | 0 | Paclitaxel Liposome 240mg+Oxaliplatin 150mg+5Fu3750mg | 6 | total gastrectomy | Oxaliplatin(320mg)+Letitrexed(4.8mg)+Lobaplatin(80mg) | Death | 1140 |
| 7 | 56 | Female | 0 | 0 | Oxaliplatin 180mg+Tegafur 60mg | 3 | total gastrectomy | Lobaplatin(100mg) | Death | 1031 |
